# Nothnagel Syndrome Without Extremity Symptoms: A Case Report

**DOI:** 10.7759/cureus.74346

**Published:** 2024-11-24

**Authors:** Gavin S Zanella, Branden Brooks, Forshing Lui

**Affiliations:** 1 Neurology, University of California (UC) Davis Medical Center, Sacramento, USA; 2 Clinical Sciences, California Northstate University College of Medicine, Elk Grove, USA

**Keywords:** diabetes mellitus, neurology, neuro-ophthalmology, physical exam, stroke, third nerve palsy

## Abstract

The finding of pupil-sparing third nerve palsy is synonymous with diabetic third nerve palsy in the minds of many clinicians. While this is the most common cause of a third nerve palsy with normal pupillary response, it is not the only cause. We present the case of an elderly diabetic gentleman who presented with pupil-sparing third nerve palsy and gait abnormalities without any weakness or incoordination in the extremities. He was initially diagnosed with a third cranial nerve mononeuropathy due to poorly controlled type 2 diabetes but was later found on MRI to have a small ischemic stroke in the dorsal midbrain. This case highlights the importance of a thorough neurological examination and the findings in such an exam that should prompt clinicians to consider etiologies other than diabetes in patients presenting with a pupil-sparing third nerve palsy. As discussed below, failure to recognize these differential diagnoses can result in poor outcomes for patients.

## Introduction

The oculomotor and Edinger-Westphal nuclei are two closely associated collections of gray matter located in the medial midbrain [1]. The third cranial nerve is composed of nerve fibers originating from the oculomotor nucleus and is tightly accompanied en route to the orbit by parasympathetic fibers originating from the Edinger-Westphal nucleus [1]. A pupil-sparing third nerve palsy occurs when a lesion destroys the nerve fibers originating from the oculomotor nucleus while having no impact on the parasympathetic fibers originating from the nearby nucleus of Edinger-Westphal [1]. The oculomotor nucleus supplies the medial rectus, superior rectus, inferior rectus, inferior oblique, and levator palpebrae superioris muscles, whereas the nucleus of Edinger-Westphal has its effects on the ciliary and constrictor pupillae muscles via the parasympathetic tone it carries [2]. Lesions that impact the function of the oculomotor nucleus and its peripheral fibers are characterized by an inability to adduct, infraduct, and supraduct the affected eye as well as a failure to elevate the eyelid [2]. If the fibers originating from the nucleus of Edinger-Westphal are also affected, the patient will have an inappropriately dilated pupil. The most common etiology of a pupil-sparing third nerve palsy is a diabetic microvascular lesion to the peripheral nerve fiber itself [3]; however, the presence of certain clinical features should prompt an investigation for less common etiologies. The alternative causes that we will discuss include posterior communicating artery aneurysm, vasculitis (i.e., giant cell arteritis), and the dorsal midbrain stroke syndromes of Claude, Benedikt, Weber, and Nothnagel. We herein present a particularly interesting case of a midbrain stroke, Nothnagel syndrome, without extremity findings.

## Case presentation

**Figure 1:**
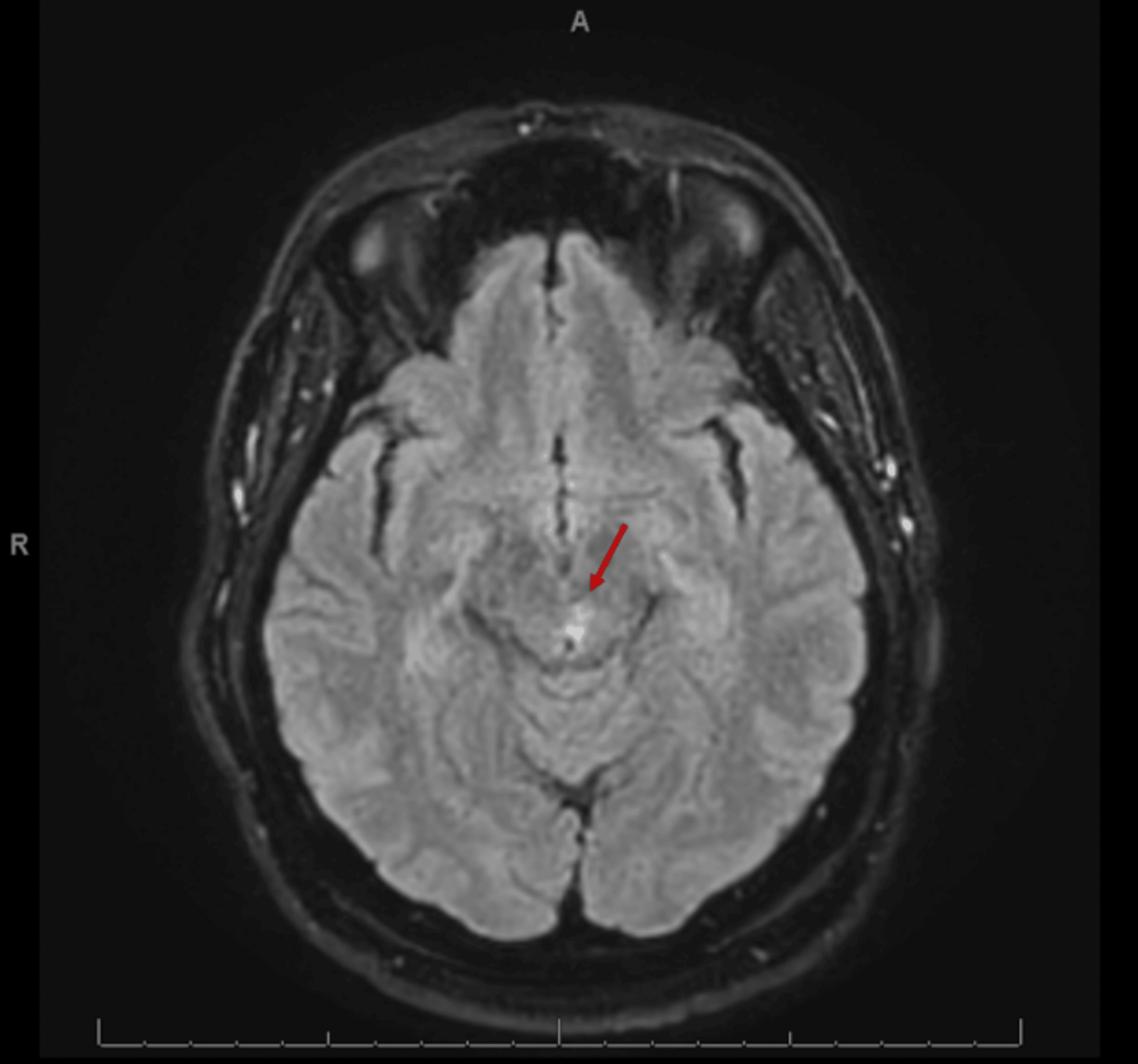
T2 flair hyperintensity of the left dorsal midbrain

Our patient is a 66-year-old right-handed gentleman with type 2 diabetes, hypertension, and hyperlipidemia who initially presented to the emergency department three weeks prior to our encounter complaining of one day of left eye drooping and unsteady gait. He was evaluated by a neurologist who noted ptosis and restriction of medial, downward, and upward gaze in the left eye, as well as the need for a walker to ambulate. The remainder of a comprehensive neurological exam, including strength, sensation, and coordination testing, was normal. Diabetic third cranial nerve mononeuropathy was cited as the most likely diagnosis, but he was advised to undergo an MRI and MRA for further evaluation. The primary team followed this plan and sought an additional consultation by ophthalmology to confirm pupillary sparing and rule out giant cell arteritis. The ophthalmologist concurred with the neurologist and stated that giant cell arteritis was highly unlikely based on their examination. The patient was discharged with a diagnosis of diabetic cranial nerve three mononeuropathy without further stroke work-up or secondary stroke prevention. Subsequently, the MRI was completed and revealed a small, recent left dorsal midbrain infarct (Figure 1). MRA was also performed and was normal.

Following this admission, the patient felt his symptoms were slowly improving until two weeks post-discharge when he began to notice his deficits becoming worse and decided to return to the hospital. At that time, a cranial nerve exam revealed left eye ptosis as well as medial, upward, and downward gaze restriction in the same eye. A careful gait examination revealed a wide-based, unsteady gait with dysfunctional turning and an inability to tandem walk. The remainder of a complete neurological exam, including cranial nerves, strength, sensation, and extremity coordination testing, was normal once again. An MRI was repeated and revealed the same lesion in the dorsal midbrain with no evidence of a new stroke. The patient’s clinical exam findings, along with the location of his lesion on MRI, were consistent with a diagnosis of Nothnagel syndrome. He was admitted for recrudescence of his subacute ischemic stroke and underwent a stroke workup. He was started on three weeks of dual antiplatelet therapy with aspirin and clopidogrel, followed by indefinite monotherapy with clopidogrel. He was discharged to a skilled nursing facility for rehabilitation.

## Discussion

This case illustrates that there are certain “red flag” findings on physical exams and imaging that suggest an etiology for pupil-sparing third nerve palsy other than diabetic ischemic neuropathy (Table 1). In the absence of any such alarm signs that will be discussed below, diabetic third nerve palsy would be the most likely diagnosis. The pathophysiology of many of the complications of poorly controlled diabetes lies in its effects on the microvasculature, and diabetic third cranial nerve mononeuropathy is no exception. The anatomy of the third cranial nerve is such that the oculomotor fibers lie in the center and the parasympathetic fibers are located superficially [1]. This arrangement leaves the deeper motor fibers more susceptible to microvascular ischemia than the superficial parasympathetics, leading to a pattern of third nerve palsy with preserved pupillary function. It is important to note that a partial cranial nerve three palsy from any cause may have pupillary sparing. We may presume that an ischemic etiology such as diabetes is causing a pupil-sparing third nerve palsy only if the palsy is complete.

**Table 1:** Etiologies and pertinent exam findings in the differential diagnoses of pupil-sparing third nerve palsy

Etiology	“Red flag” findings	Notes
Diabetes	None	Presents with a complete cranial nerve three palsy
Nothnagel syndrome	Ipsilateral ataxia	Involvement of superior cerebellar peduncle and cranial nerve III fascicle
Claude syndrome	Contralateral ataxia +/- rubral tremor	Involvement of red nucleus, superior cerebellar peduncle, and cranial nerve III fascicle
Benedikt syndrome	Hemiparesis and ataxia +/- tremor +/- choreoathetosis	Involvement of cerebral peduncle, red nucleus, and cranial nerve III fascicle in the ventral midbrain
Weber syndrome	Contralateral hemiparesis	Involvement of cerebral peduncle and cranial nerve III fascicle in the ventral midbrain
Posterior communicating artery aneurysm	Initial pupillary sparing with progression to pupillary involvement	Risk of progression to rupture and subarachnoid hemorrhage if untreated
Vasculitis	Varies; systemic symptoms and vascular involvement of other organ systems	None

In the midbrain tegmentum, the third cranial nerve fascicle is formed by its components from the oculomotor nucleus and the Edinger-Westphal nucleus. Any small lesion in the midbrain tegmentum may affect the fibers from the oculomotor nucleus yet sparing those from the Edinger-Westphal nucleus, resulting in the pupil-sparing third nerve lesion. An example of this is the syndrome that afflicted our patient, the dorsal midbrain stroke syndrome of Nothnagel. Patients presenting with this syndrome are expected to demonstrate a third nerve palsy due to involvement of the third cranial nerve fascicle as well as ataxia due to involvement of the superior cerebellar peduncle [4]. The unusual finding that originally confounded a correct diagnosis in our patient was the absence of any extremity findings. It was only after performing a gait exam that his ataxia could be identified. This provides an excellent argument for the importance of a deliberate gait evaluation during every neurological exam, in which tandem walking is the most sensitive test for ataxia [5]. Our patient’s presentation can likely be explained by the involvement of the pedunculopontine nucleus or its exiting fascicles, a medial midbrain structure that has been implicated in the development of gait ataxia when damaged [6]. His particular lesion did not impact the cerebellar peduncles, and thus he did not present with extremity ataxia.

Next, we will discuss the midbrain stroke syndromes of Claude, Weber, and Benedikt. A patient presenting with Claude syndrome would be expected to have a third nerve palsy, again due to a fascicular lesion, as well as contralateral ataxia and rural tremor. The latter two of these three symptoms are classically thought to be due to lesioning of the red nucleus [7]. However, more recent studies suggest that lesions resulting in Claude syndrome occur just inferomedial to the red nucleus, catching the efferent fibers of the cerebellum [7,8]. Weber syndrome presents with cranial nerve three palsy and contralateral hemiparesis due to the involvement of the cerebral peduncle [9]. This rare stroke syndrome is not uncommonly reported as occurring with pupillary sparing [10]. The last of the midbrain syndromes to consider is Benedikt syndrome, which presents with cranial nerve three palsy, contralateral ataxia (due to damage of the red nucleus), and contralateral weakness (due to damage of the cerebral peduncle) [11]. This syndrome may also present with Holmes tremor and/or choreoathetosis [11]. Any of these syndromes can present with pupillary sparing as the lesions often impact the nerve fascicle, and therefore, the presence of any of the focal neurological deficits listed above along with a pupillary sparing third nerve palsy should prompt an investigation for stroke.

Another possibility, which was investigated during our patient’s original hospitalization, is vasculitis [12,13]. The pathophysiology is similar to diabetic third nerve palsy, with disruption occurring in the microvascular supply to the third nerve, which disproportionately affects the deeper oculomotor fibers. However, in vasculitis, vascular disruption occurs due to an inflammatory process rather than vasculopathy secondary to chronic hyperglycemia. The red flag findings in these cases will vary depending on the specific vasculitis, but systemic symptoms and the presence of other vascular lesions should raise one’s suspicion.

The final differential that we will discuss is the posterior communicating artery aneurysm. Based on our previous discussion of the anatomy of the third cranial nerve and its associated parasympathetic fibers, we would expect compressive lesions to impact the parasympathetics before even reaching the oculomotor fibers. However, posterior communicating artery aneurysms may present with a pupil-sparing third nerve palsy in up to 14% of cases [14]. This is speculated to be due to the fact that in the region of the posterior communicating artery, the parasympathetic fibers are concentrated in the superior aspect of the nerve, and most aneurysms are directed inferiorly, laterally, or posteriorly [15]. A patient who initially presents with a pupil-sparing third nerve palsy and later develops pupillary involvement should prompt investigation for an aneurysm. This is an especially important etiology to identify, as neurosurgical evaluation is warranted to prevent rupture and subarachnoid hemorrhage [16].

## Conclusions

A pupil-sparing third nerve palsy is most commonly caused by but is not pathognomonic for a peripheral diabetic microvascular lesion. A careful and detailed neurological exam is always important in any patient presenting with a focal neurological deficit. This case particularly highlights how the absence of a careful gait exam led to a missed diagnosis of the Nothnagel midbrain stroke syndrome. If a neurological exam reveals the presence of the clinical features discussed above, this should prompt investigation of alternate diagnoses, including midbrain stroke syndromes, vasculitis, and posterior communicating artery aneurysm. Identification of the correct etiology is critical to ensure the best outcomes for patients, and our case is a key example of the dangers posed by a missed diagnosis.
